# Stabilizing Additives Added during Cell Lysis Aid in the Solubilization of Recombinant Proteins

**DOI:** 10.1371/journal.pone.0052482

**Published:** 2012-12-20

**Authors:** David J. Leibly, Trang Nhu Nguyen, Louis T. Kao, Stephen N. Hewitt, Lynn K. Barrett, Wesley C. Van Voorhis

**Affiliations:** 1 Department of Allergy and Infectious Disease, School of Medicine, University of Washington, Seattle, Washington, United States of America; 2 Seattle Structural Genomics Center for Infectious Disease (SSGCID), Seattle, Washington, United States of America; New England BioLabs, United States of America

## Abstract

Insoluble recombinant proteins are a major issue for both structural genomics and enzymology research. Greater than 30% of recombinant proteins expressed in *Escherichia coli* (*E. coli)* appear to be insoluble. The prevailing view is that insolubly expressed proteins cannot be easily solubilized, and are usually sequestered into inclusion bodies. However, we hypothesize that small molecules added during the cell lysis stage can yield soluble protein from insoluble protein previously screened without additives or ligands. We present a novel screening method that utilized 144 additive conditions to increase the solubility of recombinant proteins expressed in *E. coli.* These selected additives are natural ligands, detergents, salts, buffers, and chemicals that have been shown to increase the stability of proteins *in vivo*. We present the methods used for this additive solubility screen and detailed results for 41 potential drug target recombinant proteins from infectious organisms. Increased solubility was observed for 80% of the recombinant proteins during the primary and secondary screening of lysis with the additives; that is 33 of 41 target proteins had increased solubility compared with no additive controls. Eleven additives (trehalose, glycine betaine, mannitol, L-Arginine, potassium citrate, CuCl_2_, proline, xylitol, NDSB 201, CTAB and K_2_PO_4_) solubilized more than one of the 41 proteins; these additives can be easily screened to increase protein solubility. Large-scale purifications were attempted for 15 of the proteins using the additives identified and eight (40%) were prepared for crystallization trials during the first purification attempt. Thus, this protocol allowed us to recover about a third of seemingly insoluble proteins for crystallography and structure determination. If recombinant proteins are required in smaller quantities or less purity, the final success rate may be even higher.

## Introduction

Recombinant proteins that express only in the insoluble fraction are a significant issue for research laboratories. Between 2008 and 2011 we at the Seattle Structural Genomics Center for Infectious Disease (SSGCID) expressed approximately 3500 recombinant proteins. Thirty percent of these proteins expressed only in the insoluble fraction, with no protein apparent in the soluble fraction as determined by SDS-PAGE. Furthermore, the overall soluble protein rate of 4178 cloned genes from over 20 different genera of origin was only 57% and 62% of these were able to be purified; thus our experience is that only 35% of recombinant proteins produced in *E. coli* are sufficiently soluble and well folded to be purified in sufficient quantities for crystallography [1]. Similar results have been reported from other structural genomics centers. Berkeley Structural Genomics Center (www.strgen.org) and Northeast Structural Genomics Consortium reported 29.5% and 32.5% insoluble rates respectively. The insolubility rate for some species has been shown to be significantly different from this average. For example, efforts to determine the structure of all non-membrane proteins of *Methanobacterium thermoautotrophicum* yielded 57% soluble proteins [2] but only 18.7% of expressed proteins in *Plasmodium spp.* had the soluble expression levels needed for crystallography [3].

Typically when a protein is insoluble multiple rescue procedures may be undertaken including: refolding of denatured proteins [4], creating fusion protein constructs such as maltose binding protein [5]–[7], alternative expression systems such as cell-free expression [8] or baculovirus [9], or using constructs with either amino or carboxyl-terminal deletions [10]. Attempts to include molecular chaperone proteins [11] or decreasing culture temperatures [12] have proven effective in producing soluble recombinant protein in some instances. Expression of homologs of a protein of interest [13] or removing flexible loops or residues that affect solubility [14] has also led to enhanced solubility rates of proteins for structure determination efforts. These rescue methods involve additional effort, do not always work, and may be cost prohibitive to labs.

Williams *et al.,* [15] first noted that when insoluble protein expression is observed, proteins might possibly be secluded within inclusion bodies. Indeed, this has become the conventional paradigm; the belief that it’s not worth trying to solubilize proteins from *E. coli* which are apparently insoluble, as they are likely to be trapped within these inclusion bodies. We hypothesize a significant fraction of proteins are not found in inclusion bodies but rather are expressed as soluble proteins in *E. coli* and aggregate after cell lysis. These proteins would appear in the soluble fraction if the cell lysis buffer conditions were adjusted, whether it is by pH, ionic strength or presence of an additive.

Protein aggregation during purification also leads to solubility issues. In recent years Bondos and Bickell [16] have shown that recombinant protein aggregates can be solubilized during the purification process with various buffer conditions. With their method the recombinant proteins need to be present as aggregates in the soluble fraction, which are then disrupted by changing buffer compositions. We took a different approach, tackling the solubility issue earlier, at the cell lysis stage, with the aim to prevent the initial protein aggregation from occurring.

We describe a screening method with 144 unique lysis conditions followed by SDS-PAGE analysis of the soluble fractions to determine conditions that result in an increase soluble recombinant protein. This lysis with additive method is effective in rescuing protein that express as insoluble in *E. coli* avoiding the need to design new constructs or switch expression systems. Although this technique was developed for a high-throughput structural genomics project, it is applicable to any scale project to screen for increase in protein solubility.

## Results

We chose proteins for this screen in which the structure solution was of high priority and there was a significant solubility issue in a high-throughput protein expression screen [17]. Proteins that were considered were either fully insoluble or that had very high levels of recombinant protein expression in the total cell lysate, but a small percentage in the soluble fraction (<10%). Scientific impact of the proteins was considered when compiling the list of proteins to screen, which for SSGCID are proteins requested by the scientific community. This process resulted in 45 DNA sequence-validated proteins selected for the solubilization screen.

The additive screen was developed by first adapting the crystallization additive screen “ADDit” (Emerald Bio, Bainbridge Island, WA). This served as a logical starting point as these additives have known protein interactions. Since the reagents were originally intended as a protein crystallization screen, many were replaced. Additives removed were primarily volatile solvents, protein precipitants and chemicals known to alter the tertiary structure of proteins. Metal ions, salts, and some non-volatile organic compounds were retained. The final list of additives (Table 1) fit into one of four general categories: 1) additives that possibly serve as a ligand to allow the protein to remain in a soluble conformation, such as a metal [18], [19] or an amino acid; 2) additives that reduce protein-protein interactions (chaotropic agents) or stabilize intra-molecular bonds (kosmotropic agents) [20]; 3) additives known to affect protein stability such as charged amino acids, reducing agents, polyols and sugars, known to thermally stabilize proteins [21]–[25]; and, 4), additives that significantly altered buffer or salt conditions. 24 buffer variations screening pH, ionic strength and reducing agents were also included in these 144 conditions.

**Table 1:** Complete additive and buffer list at final concentrations.

Additives 1–40	Additives 41–80	Additives 81–120	Buffer Conditions 1–24
100 mM Ammonium Sulfate	0.1% Tween 80	0.2% Dimethylethylammoniumpropane sulfonate (NDSB 195)	25 mM MES pH 6.0
10 mM Barium Chloride	0.01% Triton X-100	0.2% 3-(1-Pyridino)-1-propane sulfonate(NDSB 201)	25 mM MES pH 6.0, 5 mM TCEP
10 mM Barium Iodide	0.01% CTAB	2.0% Benzamidine HCl	25 mM MES pH 6.0, 200 mM NaCl
10 mM Cadmium Chloride*	0.05% Lauryl Sulfobetaine	0.1% Formamide	25 mM MES pH 6.0, 200 mM NaCl, 5 mM TCEP
20 mM Calcium Chloride	0.05% Brij 56	100 mM Urea	25 mM MES pH 6.0, 400 mM NaCl
100 mM Cesium Chloride	0.05% Zwittergent 3-08	100 mM Guanidine HCl	25 mM MES pH 6.0, 400 mM NaCl, 5 mM TCEP
10 mM Cobalt chloride^#^	10 mM Triethanolamine HCl	50 mM Tricine	25 mM MES pH 6.0, 600 mM NaCl
10 mM Copper (II) chloride*^#^	10 mM Spermine	30 mM EPPS	25 mM MES pH 6.0, 600 mM NaCl, 5 mM TCEP
10 mM Gadolinium bromide*^#^	100 mM Sarcosine	30 mM Tris pH 8.0	25 mM Hepes pH 7.0
10 mM Holmium chloride*^#^	10 mM Trimethylamine N-oxide	2.5 mM SAM	25 mM Hepes pH 7.0, 5 mM TCEP
10 mM Lanthanum acetate^#^	10 mM Glycine betaine	Vitamin B12^#^	25 mM Hepes pH 7.0, 200 mM NaCl
100 mM Lithium chloride	2.0% Mannitol	10 mM Biotin	25 mM Hepes pH 7.0, 200 mM NaCl, 5 mM TCEP
100 mM Lithium sulfate	2.0% Erythritol	50 uM Riboflavin	25 mM Hepes pH 7.0, 400 mM NaCl
10 mM Magnesium chloride	5.0% Trehalose	10 mM α-Cyclodextrin	25 mM Hepes pH 7.0, 400 mM NaCl, 5 mM TCEP
10 mM Manganese chloride	5.0% Glucose	10 mM choline chloride	25 mM Hepes pH 7.0, 600 mM NaCl
100 mM Potassium chloride	5.0% Sucrose	0.5% Brij 35	25 mM Hepes pH 7.0, 600 mM NaCl, 5 mM TCEP
100 mM Potassium citrate	5.0% Xylitol	0.5% LDAO	25 mM Tris pH 8.0
5 mM Samarium bromide*^#^	1.5% β-cyclodextrin	0.5% Triton X-100	25 mM Tris pH 8.0, 5 mM TCEP
10 mM Samarium chloride*^#^	0.5% 1,2,3-heptanetriol	0.5% CTAB	25 mM Tris pH 8.0, 200 mM NaCl
50 mM Sodium fluoride	3.0% 6-aminocaproic acid	0.5% Lauryl Sulfobetaine	25 mM Tris pH 8.0, 200 mM NaCl, 5 mM TCEP
100 mM Sodium malonate	3.0% Ethylene glycol	0.5% Tween 60	25 mM Tris pH 8.0, 400 mM NaCl
10 mM Yttrium chloride*^#^	3.0% Gamma butyrolactone	0.5% Brij 56	25 mM Tris pH 8.0, 400 mM NaCl, 5 mM TCEP
10 mM Yttrium nitrate*^#^	100 mM Glycine	0.5% Zwittergent 3-08	25 mM Tris pH 8.0, 600 mM NaCl
10 mM Sodium selenite	10 mM Gly-Gly-Gly	0.5% Brij 93	25 mM Tris pH 8.0, 600 mM NaCl, 5 mM TCEP
5 mM Zinc chloride*	5.0% Jeffamine M-600^#^	0.5% Octylβ-D-glucopyranoside	
10 mM Nickel chloride*	5.0% PEG 300^#^	0.5% N-lauryl sarcosine	
10 mM Sodium molybdate	5.0% PEG 4000	0.5% ASB-14	
10 mM Iron (III) Chloride*^#^	5 mM DTT	100 mM Dipotassium phosphate	
10 mM Ammonimun nitrate	5 mM BME	375 mM L-Arginine	
10 mM Sodium thiocyanate	5 mM TCEP	0.5 M Proline	
10 mM Ammonium acetate	5 mM EDTA	1 M Glycine betaine	
10 mM Potassium nitrate	5 mM EGTA pH 8.0	1 M 3-(1-Pyridino)-1-propane sulfonate (NDSB 201)	* Additive was made in dH_2_O onlyFinal pH was not adjusted
10 mM Sodium acetate	10 mM ATP	1 M Xylitol	# Samples containing this additive must be diluted prior to SDS-PAGE analysis
0.2% ASB-14	10 mM ADP	0.5 M Mannitol	
0.05% Brij 93	10 mM GTP	0.7 M Trehalose	
0.05% Octyl β-D-glucopyranoside	75 mM L-Arginine	50 mM α-Cyclodextrin	
0.01% Brij 35	50 mM Taurine	2 M Formamide	
0.05% LDAO	5 mM Glutamic Acid	1 M Dimethylethylammoniumpropane sulfonate (NDSB 195)	
0.05% N-lauryl sarcosine	100 mM Proline	1 M Trimethylamine N-oxide	
0.1% SDS	100 mM Imidazole	100 mM Triethylamine	

**Figure 1:**
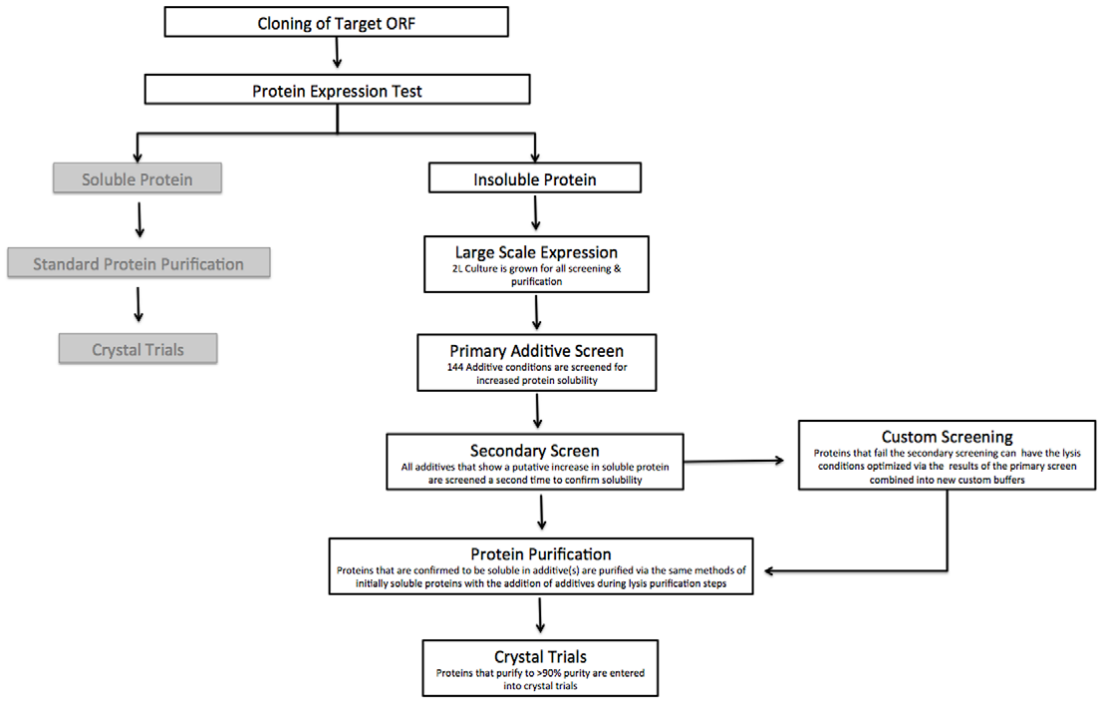
Flowchart of the additive screen in the context of high-throughput structural genomics. The target open reading frame is cloned into SSGCID vectors then expression testing is performed [25]. If the protein is soluble it is entered into our standard purification pipeline [28]. High priority insoluble proteins enter into additive screening. One two-liter culture is grown to obtain pelleted *E. coli* expressing the recombinant proteins for the screens, and then the 144 condition primary screen is conducted. All conditions that show an increase in solubility are subjected to a secondary screen for confirmation. Successful secondary screen proteins are then purified with additives and those that are >95% pure and yield >5 mg enter into crystal trials. In the event that the secondary screen fails, the results from the primary screen may be used to guide the creation of new additive and buffer combinations for custom screening.

Each protein was expressed in *E. coli* at 2-liter scale as previously published [17], [26]. This two-liter batch was first screened against all conditions in 0.5 ml lysis volumes to search for increased solubility (primary hit). Primary hits were subjected to a secondary screen to confirm solubility. Standard expression procedures and lysis with the appropriate additives allows purification under standard native conditions with no need for inclusion body preps and protein refolding [27], [28]. Large-scale purifications were attempted for 15 of the proteins and 6 were purified in sufficient quantity (>5 mg) and purity (>95%) for crystallization. The general schema of the screen is presented in *Figure 1* and further discussed in the materials and methods section. In approximately one third of the cases, the solubility observed in the screen resulted in a practical quantity of pure protein for crystallography. The screening and purification can be performed with the single 2-liter expression preparation.

Forty-five proteins initially met the criteria for the screen. Four plasmids failed to produce discernible recombinant expression when grown in large-scale, despite the initial screen suggesting insoluble protein production. After three attempts, work was stopped on these proteins. All 41 successfully expressed proteins were screened. The overall success rate of this is presented (Table 2) as well as protein specific results (Table 3). Thirty-four proteins showed increased solubility in multiple conditions; only one of these failed to remain soluble in secondary screens (Table 3). There were total of 239 primary hits between all 33 proteins (83% of proteins screened) where an additive showed some indication of increased solubility. From these 239 primary hits, 75 (31%) were soluble in the secondary screens. Thirty-two of these 33 proteins had previously appeared completely insoluble during the initial solubility screening. Thus, primary and secondary screens suggest over 80% of proteins (33 out of 41 proteins screened) can have improved solubility when lysed with additives. The recombinant proteins that had their solubility increased originated from 14 diverse species of bacterial, parasitic and fungal organisms. Analysis into the protein isoelectric point (pI) and the predicted disorder of all the protein targets selected for this study was performed with no correlation between these factors and success in the screen (data not shown).

**Table 2:** Screening step success rate.

Step	Number	Overall Success %
Proteins Selected	45	N/A
Proteins Expressed	41	100
Proteins with >2 primary hits	34	83
Proteins Soluble in Secondary	33	80
Soluble in >2 Additive Conditions	16	39

**Table 3:** Individual protein screening and scale-up results.

Species	Protein	Uniprot ID	Screening and Scale-upResult	Solubilization Additive
*Ajellomyces capsulata*	Lanosterol 14-alpha-demethylase	Q68HC5	Failed to express	
*Aspergillus fumigatus*	14-alpha sterol demethylase Cyp51B*	Q96W81	Solubilized	Trehalose
*Aspergillus fumigatus*	14-alpha sterol demethylase Cyp51A	Q4WNT5	Screen failed to yield soluble	
*Borrelia burgdorferi*	Sensory transduction histidine kinase, putative*	O51381	Solubilized, purified, crystal trials	Trehalose, Mannitol
*Burkholderia pseudomallei*	Sensor protein*	Q3JG94	Solubilized, purified, crystal trials	Trehalose
*Burkholderia pseudomallei*	Sensor protein *	Q3JS34	Solubilized, purified, crystallized, did not diffract	Trehalose
*Burkholderia pseudomallei*	Pentapeptide repeat family protein*	Q3JL51	Solubilized	Trehalose, L-Arginine
*Burkholderia pseudomallei*	Metallopeptidase domain protein*	Q3JGM7	Solubilized, self-cleaving, purified, crystal trials	Trehalose
*Candida albicans*	Lanosterol 14-alpha demethylase	Q9UVT4	Screen failed to yield soluble	
*Candida glabrata*	Lanosterol 14-alpha demethylase	P50859	Screen failed to yield soluble	
*Coccidioides immitis*	Cytochrome P450 51	E9DGX7	Failed to express	
*Coccidioides immitis*	Cytochrome P450 51*	E9DIY7	Solubilized	Trehalose
*Coccidioides posadasii*	Metalloprotease 1*	Q71H76	Solubilized	Xylitol
*Coccidioides posadasii*	Proline-rich antigen 2*	Q6K1L8	Solubilized, purified, yielded diffracting protein crystals, but structure not solved yet	Potassium Citrate
*Coccidioides posadasii*	Proline-rich antigen 5*	Q3Y5I2	Solubilized, purified, crystal trials	Trehalose
*Cryptococcus neoformans*	Lanosterol 14 alpha-demethylase*	Q09GQ2	Solubilized, purified, crystal trials	Mannitol, SDS
*Cunninghamella elegans*	Cytochrome P450 51*	Q9UVC3	Solubilized	Octyl β-D-glucopyanoside
*Entamoeba histolytica*	C2 domain-ontaining protein*	C4M344	Solubilized	Mannitol & Potassium Citrate
*Entamoeba histolytica*	PRMT7 homologue	C4LST3	Solubilized	Samarium (III)
*Entamoeba histolytica*	Putative uncharacterized protein*	C4LX71	Solubilized	Trehalose
*Issatchenkia orientalis*	Lanosterol 14-alpha-demethylas*	Q874Q6	Solubilized	Xylitol
*Leishmania donovani*	Elongation factor 1-alpha*	Q95VF2	Solubilized	Trehalose & Glycine betaine
*Mycobacterium tuberculosis*	FADE29	P71858	Screen failed to yield soluble	
*Mycobacterium tuberculosis*	LPPN Rv2270*	Q50693	Solubilized	Trehalose, L-Arginine
*Mycobacterium tuberculosis*	Hypothetical protein Rv3172c	O53322	Failed during purification	
*Mycobacterium tuberculosis*	Uncharacterized protein Rv3683*	O69651	Solubilized	Trehalose
*Pneumocystis jiroveci*	Lanosterol 14-alpha demethylase*	Q875H2	Solubilized	Trehalose, Proline, Glycine betaine, NDSB 201, Mannitol
*Saccharomyces cerevisiae*	Lanosterol 14-alpha demethylase*	P10614	Solubilized	Glycine betaine
*Toxoplasma gondii*	Rhoptry kinase family protein ROP22*	B6KP01	Solubilized	Copper (II) chloride
*Toxoplasma gondii*	Rhoptry kinase family protein ROP1	B6KEY1	Failed during purification	
*Toxoplasma gondii*	Rhoptry kinase family protein ROP28*	B6KB67	Solubilized	Copper (II) chloride
*Toxoplasma gondii*	Rhoptry kinase family protein ROP40*	B6KL15	Solubilized	Copper (II) chloride
*Toxoplasma gondii*	Membrane skeletal protein IMC1*	B6KJM2	Solubilized	Arginine, Trehalose
*Toxoplasma gondii*	Rhoptry protein ROP7*	B6KR07	Solubilized	Trehalose, CTAB
*Toxoplasma gondii*	Malaria antigen, putative	B6KFD4	Screen failed to yield soluble	
*Toxoplasma gondii*	Surface antigen P22*	B6KD48	Solubilized, purified, crystal trials	TCEP
*Toxoplasma gondii*	Hsp20/alpha crystallin domain-containing protein*	B6KKL2	Solubilized	Trehalose, NDSB 195, Mannitol
*Toxoplasma gondii*	TgDIP13	B6KUH1	Failed to express	
*Toxoplasma gondii*	Receptor for activated C kinase, RACK protein	B6KSU1	Screen failed to yield soluble	
*Toxoplasma gondii*	Unnamed apical complex protein*	B6KDE9	Solubilized	Potassium Citrate, ASB-14, Trehalose, Glycine betaine, Proline, K2HPO4, Mannitol
*Toxoplasma gondii*	TgDCX*	B6KAS6	Solubilized	Trehalose, L-Arginine, Proline, NDSB 201, Mannitol, Formamide
*Toxoplasma gondii*	Unnamed apical complex protein*	B6K951	Solubilized	Potassium Citrate, Trehalose,
*Toxoplasma gondii*	Unnamed apical complex protein*	B6KBK7	Solubilized	Trehalose, L-Arginine, Proline
*Toxoplasma gondii*	Unnamed apical complex protein*	B6KN56	Failed to express	
*Toxoplasma gondii*	Unnamed apical complex protein*	B6K9R8	Solubilized	Trehalose, Triton X-100, CTAB

* Protein previously appeared totally insoluble.

Twenty additives contributed to this increased solubility and 11 additives affected two or more proteins (trehalose, glycine betaine, mannitol, L-Arginine, potassium citrate, CuCl_2_, proline, xylitol, NDSB 201, CTAB, and K_2_PO_4_). The number of proteins for each of these additives is presented in Table 4; molecular structures are presented in *Figure 3*.

**Table 4:** Top additives results.

Additive	Primary Hits	Soluble Protein (Secondary Screen)
0.75 M Trehalose	25	21
1 M Glycine betaine	21	4
0.5 M Mannitol	18	7
0.1 M Potassium citrate	12	4
1 M Trimethylamine N-Oxide	12	0
0.5 M Proline	11	4
1 M Dimethylethylammoniumpropane sulfonate (NDSB 195)	11	1
0.375 M L-Arginine	8	6
1 M Xylitol	8	2
0.01 M Sodium selenite	8	0
1 M 3-(1-Pyridino)-1-propane sulfonate(NDSB 201)	6	2
0.1 M Dipotassium phosphate	5	2
CuCl_2_	3	3
CTAB	3	2

Osmolytes were the top performing additives, which was expected. Osmolytes protect proteins from denaturation *in vivo* and have been studied for their *in vitro* thermal stabilizing properties. Trehalose was the top-performing additive, yielding increased solubility for 21 proteins. Trehalose is a glucose disaccharide that serves as an osmolyte and desiccation protectant in many lower life forms and has been studied for its effects on increasing protein stability and preventing protein aggregation [29]–[34]. Emerging research indicates that trehalose and trimethylamine N-oxide (TMAO) aid the folding and refolding of proteins [35]. It was hypothesized that these osmolytes could serve as chemical chaperones *in vivo* with different effects on proteins based on where disordered regions were located on a protein and were shown to change protein-folding rates.

The six-carbon polyol, mannitol aided in the solubility of seven SSGCID proteins. Polyols have been shown to thermally stabilize proteins [22], [25], [34], [36]. Two other natural osmolytes, glycine betaine (betaine) [36]–[40] and proline [38]–[42] have increased protein solubility for four proteins each, with indications of increased solubility for 21 proteins (betaine) and 10 proteins (proline) in primary screens. Arginine proved effective in solubilizing seven proteins. Arginine has been widely studied for its ability to stabilize proteins and prevent protein aggregation [22], [43]–[45] and has consistently shown prevention of aggregation of various unrelated proteins, allowing proteins to achieve higher concentrations and remain in solution compared to samples without arginine present [8]. Citric acid is an intermediate in the citric acid cycle, and a buffering agent used in the food industry. Potassium citrate was effective in solubilizing four proteins in the secondary screen. Citrate has previously shown to aid in the stabilization of several proteins [46]–[49].

Metal ions increased solubility in four instances. Three *Toxoplasma gondii* rhoptry proteins were soluble in the presence of 10 mM CuCl_2_. One *Entamoeba histolytica* protein, a protein arginine methyltransferase homologue, is soluble in the presence of samarium^3+^. Thus far, it has been difficult to further study these proteins and determine the effect the metals are have as the ions interfere with many purification methods such as metal affinity & ion exchange chromatography.

The final additives of note to solubilize significant numbers of proteins in our screen were the non-detergent sulfobetaines (NDSBs) dimethylethylammoniumpropane sulfonate (NDSB 195) & 3-(1-Pyridino)-1-propane sulfonate (NDSB 201). NDSBs are zwitterionic molecules shown to aid in protein stability and in folding [23], [50] and are commonly used as an additive in protein refolding experiments.

Overall most additives appeared to solubilize many more proteins in the primary screen than the secondary screen. When this discrepancy was observed for the first few proteins, it was assumed human error was to blame. Given the consistency of the observation, however, it seems likely that many of these proteins were soluble in the primary screens, but the solubility could not be replicated in the secondary screen, perhaps because the ratio of the protein to additive is higher in the secondary screens. For our screening protocol, one two-liter culture was grown for all screening to be done. For primary screens, 0.5 ml aliquots were taken in 96-well deep well blocks and then all the remaining cell paste was frozen and stored for secondary screens and purification. For the secondary screens frozen cell paste (approximately 0.25 g) was placed into a 15 ml conical tube and lysis buffer was added. Additives that had primary hits with some proteins that failed in secondary screens did successfully solubilize other proteins. Glycine betaine had the greatest number of false positive primary screens (17 or 81% false positive rate). The false positive rates for mannitol and xylitol were 61% and 75% respectively. Trehalose had the lowest false positive rate (16%). Experiments were done to examine the effect of increasing additive concentration for trehalose, TMAO and glycine betaine with four of the proteins (data not shown). This indicated that there was a trend of increasing additive concentration to increased protein solubility with trehalose. TMAO failed to result in any soluble proteins in the secondary screens. Since TMAO has been studied extensively for its ability to aid in protein stability [36], [39], [51]–[53], it is surprising that all 12 primary hits failed to be confirmed. Future efforts should be undertaken to determine if higher TMAO concentrations aid in reproducible solubility at the lysis step as there has been an established link established between TMAO concentration and refolding rates [35].

**Figure 2:**
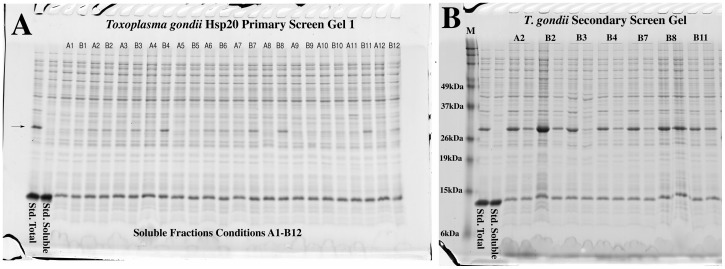
SDS-PAGE gels from the screen of *T. gondii* Hsp20. (A) Presented is one of the six SDS-PAGE gels from the primary screening experiments of Hsp20 from *Toxoplasma gondii.* Six screening gels are performed per protein to screen all 144 unique cell lysis conditions, 24 conditions per gel. Each gel is run with the protein-lysed apo (without additives) on the far left side of the gel. “Std. Total” is total cell lysate, lysed without additives. “Std. Soluble” is the soluble fraction of the non-additive lysed cells. The lanes between ‘A1’ and ‘B12’ are the 24 conditions screened on this gel. The expected molecular weight of the recombinant protein, *Toxoplasma gondii* Hsp20, is indicated with the arrow. The conditions that appeared to increase solubility and were subsequently re-screened are LDAO (A2), L-Arginine (B2), L-Proline (B3), Glycine Betaine (B4), Mannitol (B7), Trehalose (B8), NDSB 195 (B11), and Trimethylamine N-Oxide (B12). (B) SDS-PAGE gel image shows the individual secondary screen for the *Toxoplasma gondii* Hsp20 from figure 1A. The *Toxoplasma* Hsp20 protein has an ‘Apo’ control that was lysed without any additives present. Additive conditions are in the lane to the right of each Apo. Lanes marked ‘T’ are the total cell lysate, lanes marked ‘S’ are the soluble fraction for each condition. The overexpressed protein band at ∼28 kDa is the protein of interest. Conditions B8 (Trehalose) and B11 (NDSB 195) proved to be the best in solubilizing the protein, where close to 100% of the protein was present in the soluble fraction when compared to the total cell lysate, as opposed to conditions B2–B7 where there is a clear distinction between the relative amount of recombinant protein present in the soluble fraction compared to the total fraction.

The screening result of one protein is presented in *Figure 2*. Hsp20 from *Toxoplasma gondii* initially screened as insoluble in the expression testing. When screened in the primary screen seven additives showed a possible increase in protein solubility (*Figure 2A* *)* the additives were LDAO (A2), L-Arginine (B2), L-Proline (B3), Glycine Betaine (B4), Mannitol (B7), Trehalose (B8), NDSB 195 (B11), and Trimethylamine N-Oxide (B12). These additives were selected for the secondary screen, although in many cases additional additives showed an apparent band at the correct molecular weight of Hsp20, these were deemed to be the best possible candidates due to the intensity of the band on the SDS-PAGE gel. *Figure 2B* shows the secondary screen SDS-PAGE gel. Here all seven additives were re-screened and compared the cells lysed in buffers containing no additives. All cell paste samples used here, as well the primary screen, originated from the same two-liter expression culture. When compared to the non-additive control, all conditions with additives show an increase in soluble Hsp20 levels. L-Proline (B3) showed the least increase in soluble protein and Trehalose (B8) the most based on band intensity. After the success of the secondary screen the protein was purified with 750 mM (∼26% w/v) trehalose in the lysis buffer, 150 mM (5% w/v) in immobilized metal affinity chromatography buffer and 30 mM (1% w/v) in the size exclusion chromatography and final crystal trial buffer. The *T. gondii* Hsp20 was purified to sufficient purity for crystal trials. At the time of this publication the protein has not crystallized.

**Figure 3:**
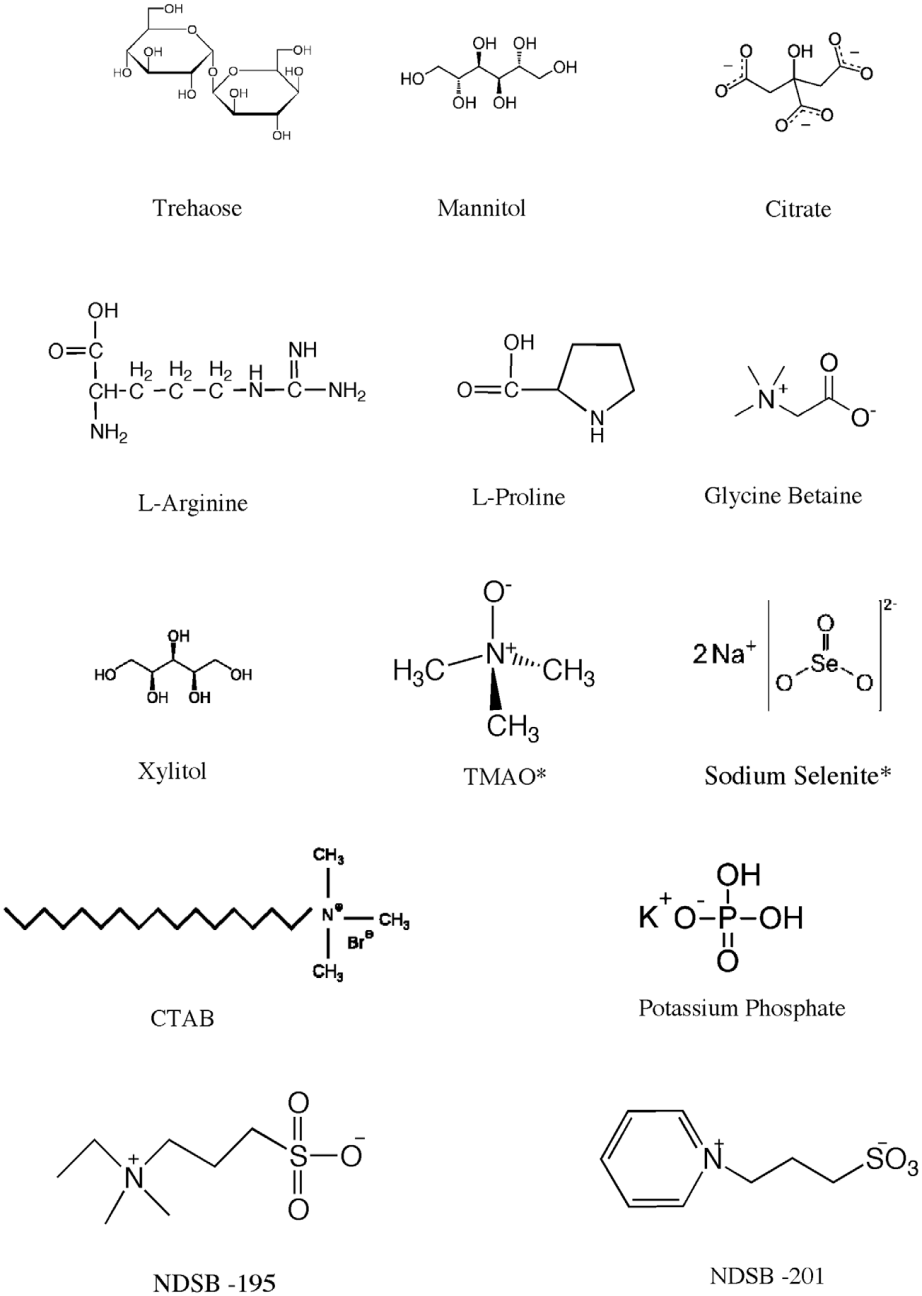
The top performing additive molecular structures. Presented are the molecular structures of the top performing additives. The number of proteins soluble with each additive is presented in table 4.

We created custom buffers to attempt to rescue proteins that did not re-screen as soluble in the secondary screens. Primary screen additives were combined to form seven buffers, two of which resulted in soluble protein. The first example is a C2 domain-containing protein from *Entamoeba histolytica*. The protein was soluble in a buffer containing potassium citrate and either mannitol *or* trehalose (Table 4). The second protein was EF-1α from *Leishmania donovani.* The additives and buffer conditions that appeared to lead to improved solubility in the primary screens were combined into a custom lysis buffer that resulted in soluble protein. This buffer contained glycine betaine, trehalose and a lower NaCl concentration (Table 4). These successes lead us to believe that many more proteins that fail to be soluble in one additive in the secondary screen can be solubilized in a predicted custom buffer derived from these primary additive hits. Published results have shown that varying mutated amino acids, and where these mutations are located on a protein, leads to preferential stabilization via different osmolytes [35]. This could suggest that the two osmolytes were necessary to stabilize different sites on the EF-1α protein.

Of the 33 proteins demonstrating increased solubility with additives confirmed with the secondary screen, large-scale purifications have been attempted on 15. Two proteins failed to solubilize when scaled up for a full purification, the other 13 proteins were recovered in the soluble fraction. With the two proteins that failed upscale solubilization, all the recombinant protein was seen in the insoluble fraction despite being lysed with the same additive that led to optimal solubility in both the primary and secondary screens.

In the remaining 13 cases, the cells were lysed in the concentration of additive from the screen, then the additive concentration was reduced during gel filtration purification; in all 13 cases the proteins remained soluble in the reduced additive concentration. Proteins that screened soluble with trehalose the cells were lysed in 750 mM trehalose, this was reduced to 30 mM (1% w/v) in the gel filtration buffer, and this 30 mM trehalose buffer was subsequently used for crystallization trials. None of these proteins suffered from precipitation issues during the purification. The concentration of trehalose was reduced in an attempt to reduce the chances of the additive affecting subsequent crystallization trials and limit to cost. Although 13 proteins in total yielded some soluble protein, only eight have achieved the final purity (>95%) and quantity (>5 mg) needed for crystal trials (Table 3).

**Figure 4:**
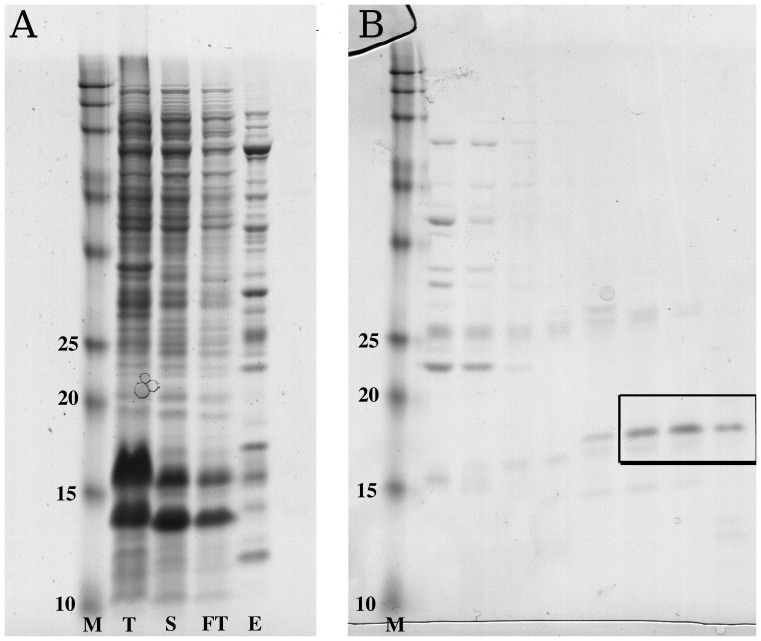
SDS-PAGE of the purification of *Coccidioides posadasii* Proline-Rich antigen 2 (Prp2). SDS-PAGE gels from Prp2, which was purified in the presence of 100 mM potassium citrate. The resulting protein resulted in diffraction quality crystals. On the left are samples from the metal affinity purification step. Lanes are as follows: “M” molecular weight standards with the corresponding weights in kDa indicated; “T” total cell lysate; “S” soluble cell lysate after centrifugation; “FT” flow through from the affinity purification column; “E” eluate from the affinity column. The band corresponding to Prp2 is marked the arrow “P”, the arrow “L” is lysozyme added during lysis. Select size exclusion chromatography fractions were analyzed via SDS page, the fraction deemed the purest concentrated for crystal trials are boxed. This protein formed diffracting crystals but the structure has not yet been solved.

At the time of this publication, two of these proteins, a sensor protein of *Burkholderia pseudomallei* (UniProt ID Q3JS34), and *Coccidioides posadasii* Proline-rich antigen 2 (Prp2) (Uniprot ID Q3Y5I2), have produced crystals. Unfortunately, in the first case the crystals failed to diffract and in the second case, the crystals diffracted but structure determination could not be completed. In both cases the crystal growth conditions are being optimized. Crystallization of this protein is significant; it indicates that these proteins are properly folded and homogeneous in solution after lysis with additives. SDS-PAGE gels from the purification of Prp2 are presented in *Figure 4*. Prp2 was first enriched from the cell lysate via a metal affinity chromatography column; in this case the column eluate was very heterogeneous. With further purification over a size exclusion chromatography (SEC) we were able to achieve sufficient purity for entry into crystal trials. Fractions from the SEC that were the most pure were pooled together and concentrated to 13.5 mg/ml with no precipitation.

A third protein, a putative *Burkholderia pseudomallei* metallopeptidase (UniProt ID Q3JGM7), was successfully solubilized in the screen and further showed activation via self-cleavage. Homologues of this protein in *Burkholderia cenocepacia* have been previously studied and have shown a characteristic auto-activation that was also observed with our homolog [35]. This protein is expressed as a zymogen that must undergo auto-cleavage to obtain the mature enzyme. The protein from this screen expressed as an insoluble protein of ∼60 kDa when screened, it was found to be soluble in trehalose. During the purification of this protein maturation of the protein was observed resulting in a ∼30 kDa protein after gel filtration which was subjected to crystallography trials (outcome still pending at the time of this publication). Properly folded protein is needed for this activation and is an indication that this protein is properly folded when solubilized in this screen, further validating this solubilization method and indicating that proteins that appear insoluble can be properly folded in the expression *E. coli*.

In an attempt to determine if inclusions bodies of recombinant protein were present in our *E. coli* expressing 12 of the recombinant proteins described here, phase contrast microscopy was performed. Refractile intracellular bodies, similar to those described as inclusion bodies in the literature [54], were not observed. However, we lacked positive controls with refractile intracellular bodies, so we cannot definitively determine the absence of inclusion bodies in our expression strains.

## Discussion

**Figure 5:**
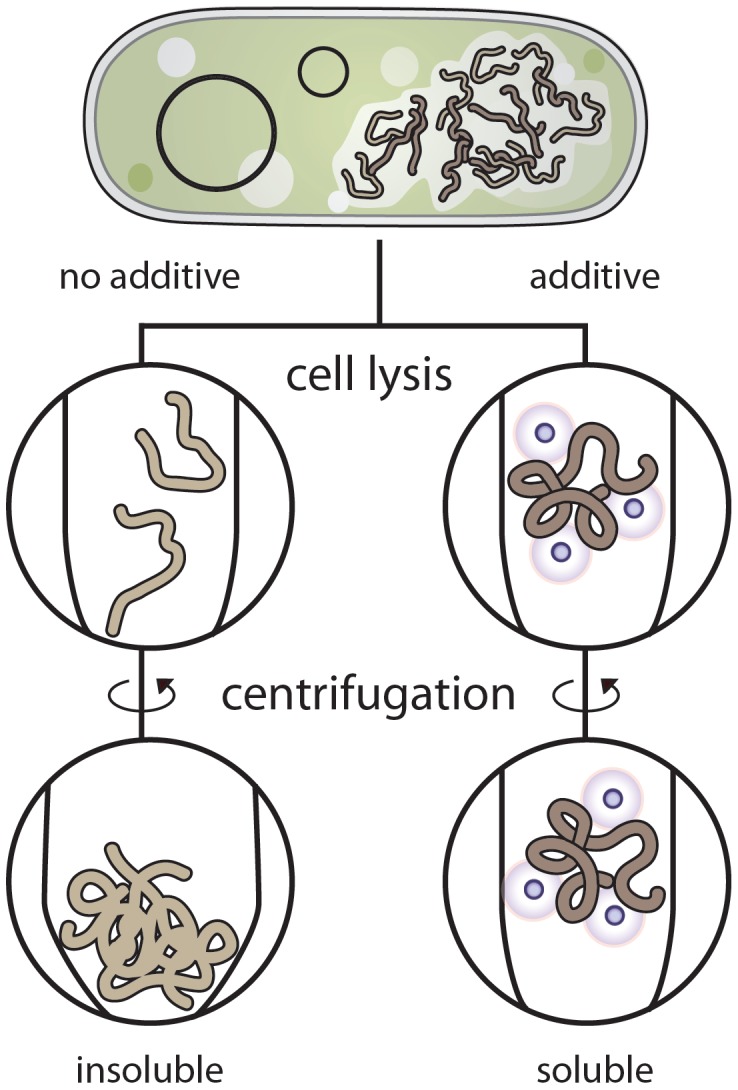
Proposed mechanism for rescue of recombinant protein solubility. We hypothesize that up to 80% of the seemingly insoluble recombinant proteins are in a partially folded state and reside in the *E. coli* cytosol. If lysed in a non-ideal buffer, the proteins unfold, resulting in aggregates of insoluble protein. When the sample is centrifuged to separate the soluble fraction, the protein aggregates are present in the insoluble cell pellet. If the additives are present during cell lysis, they can either stabilize the proteins from partially unfolding, preventing protein-protein interactions, or aid as chemical chaperones, leading to the properly folded and non-aggregated state. When centrifuged there are minimal protein aggregates and the recombinant protein remains in the soluble fraction.

Key additives were identified that promote increased solubility for multiple proteins. Based on our observations, additives increase protein solubility in about 80% of the proteins tested in the primary and secondary screens. Thus, we believe many of the proteins subjected to our screen were initially soluble in *E. coli* and the additive stabilized this solubility (*Figure 5*). It is unlikely that SSGCID’s general lysis buffer (25 mM HEPES pH 7.0, 500 mM NaCl, 10% w/v Glycerol, 0.025% w/v NaAzide, 0.5% w/v CHAPS, 10 mM MgCl2, 0.1% w/v Lysozyme, 25 U/ml Benzonase) used for the initial protein expression screening is an ideal solubility buffer for all proteins. Thus, in some cases, our general lysis buffer likely leads to protein aggregation. Additives, when present in the lysis buffer, may be stabilizing the protein so as not to unfold and aggregate (*Figure 4* *)*. The diversity of species of origin and function of the solubilized proteins indicates these methods can be applied to almost any recombinant protein that appears insoluble.

Based on comparisons of the total cell lysate and soluble fractions, it appears that close to 100% of the overexpressed recombinant protein can be solubilized when the cells are lysed in the presence of additives; this is based on band intensity when the SDS-PAGE gels are scanned on a densitometer. Ann example this dramatic solubilization can be observed in additive lanes B8 and B11 of *Figure 2B*.

Though we did not test this, it may be beneficial to have additives present in both the growth medium of the *E. coli* and also during cell to aid in the overall solubility of the protein. If this was to be attempted, the investigator would need to ensure the additive is not toxic to the *E. coli* expression strain, not metabolized, and the solubilizing effect is scalable to growth cultures with different expression volumes. Variations to this have previously be attempted [37], [53], but not with a wide range of proteins.

Our results indicate naturally occurring osmolytes glycine betaine, proline, trehalose, and mannitol can effectively aid in the stability and solubility of recombinant proteins. Many of these additives are osmolytes, which are protein-stabilizing molecules and chemical folding chaperones in nature. Research groups are actively studying these molecules to elucidate their stabilizing effects *in vitro*. Most of these studies look into the biophysical stabilization and thermal protection of proteins. Little work has been done into applying these to stabilizing small molecules to protein solubilization. It has been recently reported that when added to a growth culture, trehalose and sorbitol (6-carbon polyol) helped two previously insoluble proteins appear in the soluble fraction [37]. In Prasad *et al.* [37] protein solubility was increased, but the protein did not appear to be fully soluble.

The general mechanism for stabilization with osmolytes is believed to be through changing the protein hydration by exclusion from the hydration layer of the protein [30], [41]. The resulting change in the protein hydration increases the energy needed to denature proteins [30]. Osmolytes have been shown to increase the thermal stability of proteins resulting in a shift in the protein’s melting temperature when present in the protein buffer [30]. Other, less common osmolytes, found in nature solubilized proteins in our screen as well. Glycine betaine; a zwitterion molecule containing a quaternary amine along with a carboxylic acid; has been found in molar concentrations in cells exposed to osmotic stress and suppresses protein aggregation [37]. Proline can be found in high concentrations in plant cells that have been exposed to stress [40].

Due to our observation of increased protein stability there may be two possibilities for the additives to increase the protein solubility, either prevention of aggregation via stabilization or assistance to the stable folded state. It has been thought that proteins naturally will exist in equilibrium between folded and unfolded states with the folded state representing the lowest energy state. Hence the equilibrium is shifted to the folded state and the energy needed to unfold a protein (ΔG) varies with every protein. If the ΔG is sufficiently low, then there will be significant protein in the unfolded state at the lysis temperatures. Stabilization via osmolytes may increase the ΔG of unfolding, such that the protein remains folded during lysis. This is consistent with our finding of additives that thermally protect proteins aid insolubility Proteins have remained soluble after drastic reduction in osmolyte concentration (∼26% w/v to 1% w/v) suggesting the beneficial effect may be maximal during cell lysis and early purification steps. These osmolytes may be stabilizing the proteins during lysis as the proteins are subject to the differences between the *E. coli* cytoplasmic solute and protein concentrations, pH and ionic strength and that of the lysis buffer rapidly once the cell is lysed. This would be similar to the way osmolytes protect proteins during times of ionic stress.

New research by Bandyopadhyay *et al.* has shown that trehalose and TMAO assist in the folding of proteins *in vivo* acting as chemical chaperones [35]. They found that these osmolytes help to fold distinct mutant protein depending on whether the mutation is on the surface or in the core of the protein, as well as showing a clear link between *in vitro* folding efficiency and TMAO concentration. From these published results and what we have observed in this study, the second possibility of protein solubilization is the assistance of the protein to the native folded state via the additives in the lysis buffer. Since different organisms utilize different protein and chemical chaperones to aid in protein folding [35], when recombinant proteins are expressed in *E. coli* from different species of origin they may not be able to fully fold to the native conformation. These proteins may be in a partially folded state in the cytosol of the *E. coli*, and once lysed in the presence of the additives that act as chemical chaperones, they are able to adopt their native state (*Figure 5*).

At this time we are unsure how four proteins are soluble with the non-osmolytes Cu^2+^ or Sm^3+^ ions, as these metals have not been previously shown to aid in protein folding and stability. These ions may directly bind to the protein and allow proper folding or may modify residues via oxidation. Unfortunately metal ions present in buffers create issues with purification methods. SSGCID utilizes hexa-histidine tags to purify the protein via immobilized metal affinity chromatography (IMAC) [28]. Metal ions present in the protein buffer interfere with the binding of the tagged recombinant protein to the Ni^2+^ of the IMAC resin. Method development for purification of recombinant proteins in the presence of metals is underway.

Previous experience of stabilizing proteins that precipitated during purification in our lab indicated changes to buffer pH or salt concentration in some cases rescued solubility during the purification. As a result of these observations pH and salt concentration were additionally screened. However, none of the 24 buffer combinations with varied pH, [NaCl], or presence of reducing agents, resulted in increased protein solubility. This is consistent with the interpretation that the successful additives performing an active role in aiding in protein folding or prevention of aggregation, and not merely stabilizing proteins because of an unfavorable buffer. Although some proteins may need to be in buffers of correct ionic strength and pH to remain soluble, this did not appear to be a factor for our set of proteins.

Although only two proteins have crystallized to date, just 15 of 33 have been attempted in large-scale purification, with eight meeting the crystallization criteria. This success further validates our additive screen for use in structural biology, as these two crystallization successes are examples of the additive’s presence not adversely affecting the crystal trial. It would be useful to validate that an additive does not negatively affect crystal growth of control proteins that are known to crystallize, prior to its wide spread use for purification for crystal trials. The seven proteins that failed to yield enough protein for crystal trials were proteins with low overall expression levels. During the initial IMAC step the protein yield was either too low or the eluate was contaminated with *E. coli* proteins and could not be purified with subsequent gel filtration. This is not unique to this rescue method as failed purifications due to low expression levels are not uncommon in standard SSGCID purification. Purifications starting from larger starting material or changes in the IMAC column such as reduced column matrix volume could yield more highly purified proteins.

It should be noted that all the cell paste for the primary and secondary screening, as well as initial purification attempts came from the same two-liter culture. However, there was some variation in the amount of cell paste used in the secondary screens, approximately 0.25 g of cell paste was removed from a frozen pellet for each secondary screen since it was impossible to know how many secondary screens would be needed before the process began. During these experiments efforts were not undertaken to ensure that the same ratio of buffer to cell paste was used between primary and secondary screening. The cell paste mass was not determined for the primary screen and therefore the exact same ratios could not be maintained in the primary and secondary screening. Discrepancies between the primary and secondary screens may result from a flaw in the screening strategy that did not ensure the same ratio of additive to protein between the primary and secondary screens. This might explain why several proteins that were soluble in the primary screen were not soluble in the secondary screen and is consistent with concentration dependent effects of osmolytes [53]. Success rates during the screening process were high enough that we did not need to correct the potential discrepancies in cell paste and additive ratios between the screens. Published results [35] show additive concentration dependent stabilization of proteins and we have seen indications that there is an effect of concentration of trehalose on stability (data not shown). Although all cell paste for the screening process originated from one culture, we did not attempt to precisely standardize the ratio of protein to additive in the experiments. The goal of these experiments and our screen was to develop a high throughput method to screen proteins for increase solubility of additives. The two screen method probably also eliminated human mistakes that occur with large-scale screens that require lots of sample manipulation.

Our results indicating that known osmolytes prove to be the best additives for solubility are not surprising; nature has selected these small molecules to protect proteins *in vivo*. The sudden shock to proteins during the lysis of the *E. coli in vitro* may be similar to the shock that proteins experience with environmental changes *in vivo*. Additional experiments are needed to understand the true mechanism of small molecule stabilization of proteins during lysis. The variety of proteins solubilized in these experiments indicates there may be a general mechanism to stability and solubility that is applicable to most recombinant proteins. Buffer supplementation with small molecule osmolytes can be a simple first step to overcome solubility issues during cell lysis, protein purification and storage of proteins from recombinant and native sources. We envision an additive screen like this to be useful when creating mutant proteins as well. When a soluble protein is rendered insoluble via point mutations, it could be quickly screen against a panel of additives as potential chemical chaperones to bring it back into the soluble fraction.

Recombinant proteins that are initially found only in the insoluble fraction need not an impassible roadblock for researchers. The screening methods and results presented here show that insoluble recombinant proteins can be rescued without dedicating significant resources. The screen’s 144 unique conditions have demonstrated 11 additives that repeatedly increased protein solubility. The successful additives (Table 4), can serve as the basis of an initial solubilization screen that would be practical for many labs to implement whether it is for structural biology or any application for recombinant proteins.

## Materials and Methods

Except where noted, all concentrated additive stock solutions were made in sterile, de-ionized water with 25 mM HEPES pH 7.0 for buffering. The additives were first allowed to fully dissolve at room temperature then the ion concentrations were adjusted. The exception is metal solutions (*) in Table 1 which did not have their pH adjusted to 7.0, when NaOH was added to increase the alkalinity, precipitation was observed. These metal solutions were made in sterile de-ionized water with no buffering agents; when diluted into the final concentration with the lysis buffer there was little impact on the pH of the lysis buffer. EGTA was made in 25 mM TRIS pH 8.0. Vitamin B12 was added to 25 mM HEPES in excess, then filtered to remove the Vitamin B12 that did not go into solution. Tricine pH 7.0, EPPS pH 7.0 and TRIS pH 8.0 solutions were made without HEPES and the pH adjusted as indicated. After all 120 stock additive solutions were made; single use amounts were aliquoted into 96 well plates. Completed plates were stored at −80°C until use. Due to the known effects of NaCl concentration and pH on protein solubility, an additional 24 buffers, varying the pH, NaCl concentration as well as the presence of reducing agents, were used to screen without the additive. These additional 24 buffers (Table 1) were concentrated in sterile de-ionized stock solution and the pH adjusted.

Of the 120 additive conditions, 24 additives were screened at two different concentrations. Two concentrations were used due to solubility issues if made in a high concentration stock solution. This allowed us to screen with sugars, polyols and detergents at ideal concentrations. Overall, 144 conditions were used in this screen: 24 unique buffer conditions and 120 additive conditions consisting of 96 unique additives.

Twenty-five SSGCID expression constructs were cloned into the pAVA0421 vector using Ligation Independent Cloning (LIC) via SSGCID protocols [17]. One ml overnight cultures in ZYP-5052 media [17], [26] were used to determine protein expression. An additional twenty proteins were made by collaborators of SSGCID and were cloned into their own expression vectors. Thus, forty-five proteins were queued for the additive screen. All proteins had a hexa-histidine tag on the amino terminus with the expression growth and screening conditions all performed according to published SSGCID protocols [17]. Once insoluble protein expression was confirmed, 2 L cultures of ZYP-5052 auto-induction media were prepared as per SSGCID and Studier’s published protocols [17], [27].

To harvest, 500 µl of culture was aliquoted into each well of a 96 well deep block. For each screen, sets of two blocks were made for each protein. The blocks were centrifuged at 4,000×g for 20 min at 4**°**C, supernatant decanted, flash frozen in liquid nitrogen, and then stored at −80°C until the screen was performed. The remaining culture was spun in a Sorvall RC12BP centrifuge (Thermofisher, Waltham. MA) at 3,500×g for 20 min at 4**°**C, cell paste was transferred to 50 ml conical tubes, flash frozen and stored at −80°C for secondary screening and future purification. This material is also used for any necessary secondary screens. For secondary screening, the frozen cell paste was scraped with a metal spatula and placed into a 15 ml conical tube. Culture aliquots were also sent for plasmid sequence validation.

Fresh lysis buffer consisting of 25 mM HEPES pH 7.0, 500 mM NaCl, 10% w/v Glycerol, 0.025% w/v NaAzide, 0.5% w/v CHAPS, 10 mM MgCl2, 0.1% w/v Lysozyme, 25 U/ml Benzonase (EMD Biosciences, Gibbstown, NJ) was prepared in room temperature de-ionized water. This standard lysis buffer was used to screen all proteins with the 120 additives. The twenty-four buffer condition screen, with 10% w/v Glycerol, 0.025% w/v NaAzide, 0.5% w/v CHAPS, 10 mM MgCl2, 0.1% w/v Lysozyme, 25 U/ml Benzonase was added to the buffer listed in Table 1 at the final concentration used in the screen.

*Figure 1* is a flowchart of the screening strategy and was conducted as follows. A one ml pellet from each protein was screened for expression of the correct molecular weight protein [17]. Once insoluble recombinant protein expression was confirmed, the protein was queued for additive screening. A sample from the total cell lysate was collected; the lysate was centrifuged at 4000×g for 30 min at 4**°**C, then a supernatant sample was taken to represent the soluble fraction. These two samples were run on each additive screen SDS-PAGE gel *in lieu* of a protein ladder. This allowed direct comparison of soluble protein levels between apo-lysed cells and cells lysed in the presence of a particular additive (*Figure 2*).

For the additive screen one plate of additives was thawed on ice and then 50 µl of additive transferred via multichannel pipet into one of the 96 deep well blocks of thawed cell paste. To this, 450 µl of standard lysis buffer was added to each well and the pellet re-suspended by pipetting. This resulted in a final additive concentration as indicated. (Table 1) The cells were lysed on a Titer Plate Shaker (Lab-Line Instruments Inc., Melrose Park, IL.), set on maximum speed, for 60 min at ambient temperature. After lysis, the plates were centrifuged at 4000×g for 30 min at 4**°**C. Samples of the soluble fraction were taken for analysis on stained SDS-PAGE gels.

Several additives (# in Table 1) caused precipitation when mixed with SDS-PAGE dissociation buffers containing DTT and SDS resulting in blank lanes on the stained gels. This occurred with the first three proteins screened. Non-reducing dissociation buffer was attempted, however this buffer yielded gels with smears and indistinct bands. To remedy this, after cell lysis with the additives, the sample of the soluble fraction was diluted with stock lysis buffer prior to addition of the protein dissociation buffer.

Stained gels were analyzed for any increased solubility of the target protein, this was judged by increase in the protein band intensity on the SDS-PAGE gel. The primary hits were re-screened (*Figure 2*) with material from the frozen cell paste. The secondary screens were performed in individual 15 ml conical tubes by the described process, with the same final additive concentration as the primary screen. Secondary screening was used to confirm that soluble protein was indeed soluble and not a false positive or the result of a human error such as accidental re-suspension of the insoluble pellet during transfer of the soluble fraction. During the primary screening the small volume of the sample as well as the difficulty in visualizing the insoluble pellet after centrifugation resulted in the possibility of agitation of the pellet, hence the secondary screening was conducted in an individual tube where the pellet could easily be seen and only the soluble fraction was sampled for SDS-PAGE. If the protein was still present in the soluble fraction of the secondary screen the protein was queued for purification.

Final purifications were performed in accordance with the SSGCID standard purification methods described by Bryan *et al.* [28], with the inclusion of the additive that produced soluble protein during cell lysis. Each protein purified had the amount of additive reduced throughout the purification to approximately 1% w/v in the final buffers, in all cases no precipitation was observed. It was decided to reduce the additive concentration to allow the protein to be shipped in a buffer that was as close as possible to our standard protein buffer for crystallization (25 mM HEPES pH 7.0, 500 mM NaCl, 5% w/v Glycerol, 0.025% w/v NaAzide,).
